# Further Developments to Strengthen Regulation and Enforcement against Illicit Tobacco Trade in Australia

**DOI:** 10.1007/s11673-026-10648-3

**Published:** 2026-10-05

**Authors:** Michaela Estelle Okninski, Cheneal Puljević, Coral Gartner

**Affiliations:** 1 Adelaide Law School, College of Business and Law, Adelaide University, Adelaide, Australia; 2 NHMRC Centre of Research Excellence On Achieving the Tobacco Endgame, School of Public Health, University of Queensland, Herston, Australia

**Keywords:** Illicit tobacco trade, Tobacco control Australia, Public health, Tobacco endgame

## Introduction

Illicit tobacco trade has become endemic in Australian retailing environments, creating numerous issues for the Commonwealth and State and Territory Governments alike. Data recently published by the Illicit Tobacco and E-cigarette Commissioner suggest that over half of Australia’s tobacco market is now comprised of illicitly traded products, resulting in an estimated loss of excise revenue between $7.7 billion to $11.8 billion (Australian Government 2025a). Despite the exponential growth of the illicit market, in 2025, Australia recorded its lowest smoking prevalence rate at 5.6 per cent daily smoking among the population aged fourteen and over (Australian Government 2025b). This suggests that Australia’s strong tobacco control policies, which include high tobacco excise and strict tobacco product requirements, continue to be effective (Winnall and Greenhalgh 2025), with the latter recently strengthened through the enactment of the *Public Health (Tobacco and Other Products) Act 2023* (Cth)). Nevertheless, the well-established association between the illicit tobacco trade and organized crime syndicates creates an increased risk of gang-related violence in the community (e.g., more than 250 arson attacks on tobacco retailers since early 2023), with profits often used to fund trafficking of other illicit drugs (Borland et al. 2026, 3–4; Alfares and Ison 2025).

How best to respond to the issues caused by the illicit tobacco trade has been a point of contention, with some Australian political parties recently championing a reduction of the current tobacco excise of up to 80 per cent to compete with the illicit market (Armstrong 2026; Wright 2026; see also Borland et al. 2026). This is a dangerous and flawed response to a significant public health issue as high tobacco excise is directly correlated with lower tobacco consumption. Furthermore, this not only undermines Australia’s priority goals under the National Tobacco Strategy 2023–2030 (Australian Government 2023) but also likely breaches Australia’s obligations under the WHO Framework Convention on Tobacco Control (World Health Organization 2003). Currently, Australia already fails to meet the highest level of implementation of Article 6 of the WHO FCTC, which is determined by taxes comprising a minimum of 75 per cent of the retail price of the most sold cigarette brand (see WHO 2025, 160).

The purpose of this article is to summarize recent law reform measures enacted by the Commonwealth Government, the Tasmanian Government, and the Western Australian Government that target illicit tobacco trade by strengthening the legislative framework by targeting those profiting from the illicit trade.^1^

## Combating Illicit Tobacco Act 2026 (Cth)

The Combatting Illicit Tobacco Bill 2026 (Cth) was introduced into Commonwealth House of Representatives on March 26, 2026, with most of the Act commencing operation on August 29, 2026 (*Combatting Illicit Tobacco Act 2026* (Cth) s 2; Parliament of Australia, 2026). A key objective of the Act is to address the longstanding issue of lax offence penalties and sub-optimal regulation and enforcement—often attributed to the surge in illicit tobacco trade—with the Minister for Home Affairs explaining that the aim is to “rebalance the risk-to-reward calculation for criminal actors, by raising offence penalties to match the severity of the harms being caused by the illicit tobacco trade’”(Explanatory Memorandum, 2026, 2).

The Bill introduces wide-ranging amendments to the *Excise Act 1901* (Cth) (“*Excise Act*”), the *Customs Act 1901* (Cth) (“*Customs Act*”), and the *Taxation Administration Act 1953* (Cth) (“*Tax Act*”); Acts which primarily focus on the unlawful importation or unlawful domestic cultivation and manufacture of tobacco products where no excise or duty has been paid.

Under the *Customs Act*, the offence penalty for intentionally importing, conveying, or possessing tobacco products with the intention to defraud the revenue—by avoiding payment of excise—was increased from a maximum penalty of ten years’ imprisonment and/or a fine of either five times the total amount of excise avoided or $364,000 (1,000 penalty units), to a maximum penalty of fifteen years’ imprisonment and/or a fine of either thirty times the total amount of excise avoided or $4,368,000 (12,000 penalty units) (*Combatting Illicit Tobacco Act 2026* (Cth) sch 1 ss 1–4; *Crimes (Amount of a Penalty Unit) Instrument 2026* (Cth) s 5). Similarly, the penalty regime for the offence of reckless importation, conveyance, or possession of tobacco products to defraud the revenue was also substantially increased to ten years’ imprisonment and/or a fine of either twenty times the total amount of excise avoided or $2,912,000 (8,000 penalty units) (*Combatting Illicit Tobacco Act 2026* (Cth) sch 1 ss 5–6).

The *Excise Act* was also amended to significantly increase offence penalties for the unlicenced manufacture of excisable goods by introducing a separate aggravated offence for excisable goods that are tobacco. The new aggravated offence penalty for intentionally manufacturing tobacco “knowing, or being reckless as to whether, the goods are excisable goods’ without an applicable licence is 7 years” imprisonment or a fine (*Combatting Illicit Tobacco Act 2026* (Cth) sch 1 ss 7–10: *Excise Act* s 25). Consistent with the Act’s objective of raising offence penalties to match the severity of the harm caused by illicit tobacco trade, if a person is ordered to pay a fine, the Act applies two formulas to calculate the total fine payable, requiring the court to impose the greater of the two, which is either a fine of $1.82 million (5,000 penalty units) or five times the amount of excise that would have been payable. The offence penalty for the strict liability offence of manufacturing tobacco without a manufacturer’s licence has been increased to a fine of $182,000 (500 penalty units) (*Combatting Illicit Tobacco Act 2026* (Cth) sch 1 ss 7–10: *Excise Act* s 25(2), (4)).

Further to this, the *Excise Act* increases the offence penalties for persons who produce tobacco seed, plant, or leaf without a valid producer’s licence, and for licenced producers who produce tobacco seed, plant, or leaf in contravention of the Act or licence conditions (*Excise Act* ss 28, 29). Additionally, for the offences of unlawful possession, moving, or selling of excisable goods that are tobacco, an aggravated offence penalty regime has been introduced (*Excise Act* ss 117, 117 A, 117B). The Act adopts the same penalty regime for fault-based and strict liability offences outlined above—seven years’ imprisonment or a fine of either $1.82 million (5, 000 penalty units) or five times the amount of excise that would have been payable, whichever is greater, or a $182,000 (500 penalty units) fine for strict liability offences (*Combatting Illicit Tobacco Act 2026* (Cth) sch 1 ss 11–10, 37–42).

Significant amendments to the *Tax Act* were also implemented to increase offence penalties for tobacco-related offences. For instance, the civil penalty for possessing 2 kg or more of tobacco in Australia without official documentation such as a tax invoice or customs declaration is now $728,000 (2,000 penalty units), significantly increased from $36,400 (100 penalty units) (*Tax Act* sub-div 295-B ss 295–70, 295–75; *Combatting Illicit Tobacco Act 2026* (Cth) sch 1 s 54). Furthermore, other specific tobacco related offences strengthened under the *Tax Act* include offences for possessing, selling, or buying various quantities of tobacco in circumstances where there is a reasonable suspicion that excise or customs duty has not been paid (see *Tax Act* sub-div 308-A ss 308–5–308–50). For example, the offence penalty for possessing, selling, and buying between 5 to 10 kg of tobacco where there is a reasonable suspicion that excise or customs duty has not been paid has been substantially increased from a fine of $72,800 (200 penalty units) or five times the amount of excise or customs duty payable on the tobacco, whichever is greater, to two years’ imprisonment, a fine of $1,092,000 or five times the amount of excise or customs duty payable, whichever is greater, or both (*Excise Act* sub-div 308-A ss 308–20, 308–35, 308–50; *Combatting Illicit Tobacco Act 2026* (Cth) sch 1 ss 61–62, 74–75, 81–82).^2^ Furthermore, the definition of “sell” under the *Tax Act* has been expanded to include “supply” to cover conduct that is crucial to illicit tobacco trade such as giving, distributing, selling, administering, or transporting, which was not previously captured under the *Act* (Explanatory Memorandum 2026, 22).

## Recent Law Reform in Western Australia and Tasmania

On May 13, 2026, the *Tobacco Products Control Amendment Act 2026* (WA) commenced operation, introducing multiple amendments into Western Australia’s tobacco licencing legislation to target the sharp increase of illicit tobacco trade. Important amendments include inserting specific offences for the sale, supply, and possession of illicitly traded tobacco, and expanding the scope of existing offence provisions for licencees who sell illicitly traded tobacco (see *Tobacco and Other Products Act 2006* (WA) ss 19, 19 A, 30 A, 30B, sch 1 (definition of “tobacco product requirement,” “tobacco packaging requirement,” “prohibited product,” “illicit tobacco product”)).

For instance, the offence penalty for possessing 25,000 or more illicit cigarettes or 25 kg or more of illicit loose tobacco attracts a maximum penalty of fifteen years’ imprisonment and a fine of $4.2 million for an individual (*Tobacco and Other Products Act 2006* (WA) s 30B(5); *Tobacco and Other Products Control Regulations 2006* (WA) reg 57E(3)). Additionally, closure order provisions have been introduced in Western Australia permitting the regulator—or Minister for short-term orders of up to ninety days—to immediately shut down tobacco retailers and other businesses for up to seventy-two hours if they are reasonably suspected of selling or supplying illicit tobacco and the closure “order is necessary to prevent or mitigate a risk of harm to public health” (*Tobacco and Other Products Act 2006* (WA) ss 120 A, 120B). Long-term closure orders of up to twelve months can be imposed by the Magistrates Court if they are satisfied that illicitly traded tobacco has been, or is being, sold or supplied from the premises (*Tobacco and Other Products Act 2006* (WA) ss 120 C). A similar approach was also recently implemented in Tasmania, where substantial amendments to strengthen the legislative response to illicit tobacco trade were introduced (see Parliament of Tasmania, 2026, 65–66).

On July 22, 2026, Tasmania’s *Public Health Amendment (Prohibited Tobacco and Other Products) Act 2026* came into force, introducing a new suite of offences for selling and supplying illicitly traded tobacco and for possessing illicitly traded tobacco for the purpose of supplying it to another person (see *Public Health Act 1997* (Tas) pt 4 s 74LD). The Act implements a tiered penalty regime, with the penalty amount depending on whether the contravention is a first, second, or third/subsequent offence, imposing a maximum penalty for individuals of $42,600 (200 penalty units, first offence), $85,200 (400 penalty units, second offence) and $170,400 (800 penalty units, third/subsequent offence) (see *Public Health Act 1997* (Tas) pt 4 ss 74LB (definition of “prohibited smoking product,” “prohibited tobacco product,” 74LD(1), (2); Tasmanian Government, 2026). Tiered offence penalties also apply to corporations who contravene these provisions which attract a higher penalty amount (see *Public Health Act 1997* (Tas) pt 4 s 74LD(1), (2)).

Further to this, the Act also introduces short-term (ninety days) and long-term (twelve months) closure order provisions, forcing businesses to stop trading. A short-term closure order can be authorized if there is a reasonable suspicion that illicitly traded products are being, or likely to be, sold at the premises, while a long-term closure order can only be made by an appeal tribunal if it is satisfied that illicitly traded tobacco products are being, or likely to be, sold from the premises (*Public Health Act 1997* (Tas) pt 4 ss 74LF, 74LG, 74LH).

It is clear that there are differences between Western Australia and Tasmania’s preferred approach to strengthen regulation and enforcement of illicit tobacco trade, with both States adopting very different offence penalties for engaging in illicit tobacco trade. Currently, it is too early to determine if the comparatively low penalties adopted in Tasmania will be sufficient to curb the illicit market. That aside, these amendments are important and now position Western Australia and Tasmania instep with developments that have already occurred in other Australian States (see Okninski et al. 2025). Furthermore, introducing closure order provisions is a strong enforcement mechanism as it forces businesses to cease trading for a specified period, preventing them from continuing to sell harmful products and also permits authorities to investigate suspected illicit trade. Victoria is the only Australian State that does not have closure order provisions; however, this is currently being considered before the Victorian Parliament (Victoria State Government 2026).

## Conclusion

Illicit tobacco trade is a complex issue that requires a multifaceted solution through robust regulation and enforcement at all levels of government and not by dismantling evidence-based tobacco control policy. These recent developments are a step in the right direction to weed out illicitly traded tobacco and to maintain Australia’s highly successful tobacco control framework. Future policy and legislative responses aiming to reduce illicit tobacco trade would benefit from a holistic approach incorporating strong law enforcement responses (e.g., closure order provisions), robust regulatory frameworks, concentrated efforts to reduce demand for all tobacco products, and coordination across all levels of government. Such measures offer a more targeted, evidence-based response to Australia’s illicit tobacco market rather than reducing excise, which would make legal tobacco products more affordable without addressing underlying illicit supply of illicit products.

## Data Availability

Not applicable.

## Footnotes

1 For discussion of previous developments in this area see M., Okninski, C., Puljević, and C. Gartner. 2025. Targeting illicit tobacco trade in Australia: Recent Amendments to State and Territory tobacco control laws. *Journal of Bioethical Inquiry* 22(4): 775.

2 Due to word constraints, it is not possible to discuss all amendments to the *Excise Act 1901* (Cth) and the *Taxation Administration Act 1953* (Cth) introduced by the *Combatting Illicit Tobacco Act 2026* (Cth). For further discussion see *Combatting Illicit Tobacco Act 2026* (Cth) sch 1 pt 2; Explanatory Memorandum, Combatting Illicit Tobacco Bill. 2026. *Parliament of Australia* 12–18, 20–31 https://parlinfo.aph.gov.au/parlInfo/search/display/display.w3p;query=Id%3A%22legislation%2Fems%2Fr7458_ems_2f59b179-6bf2-4034-8b33-242b8fd1564c%22.
